# Supplementary material for: An altered cell-specific subcellular distribution of translesion synthesis DNA polymerase kappa (POLK) in aging mouse neurons
Source: eLife. 2026 May 13;13:RP101533. doi: 10.7554/eLife.101533 (PMC13171111; doi:10.7554/eLife.101533)
Supplement: Figure 1—source data 1. [file elife-101533-fig1-data1.zip › Figure 1_Source Data 1.pdf]

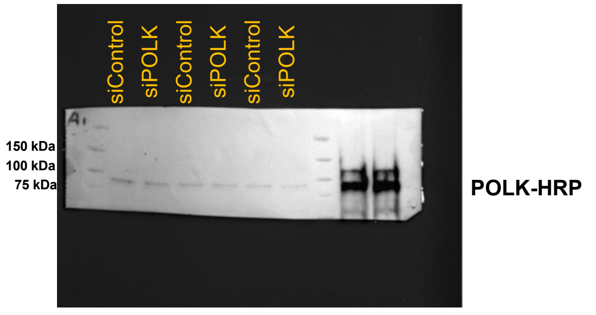

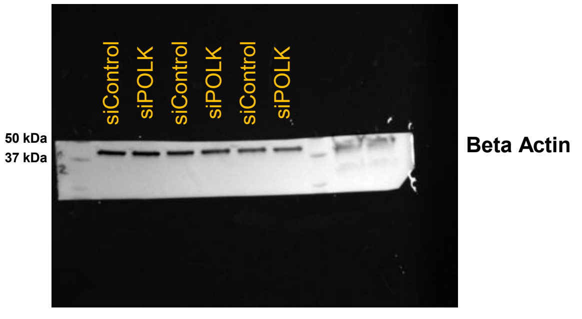

Figure 1, Source Data 1. Original blots corresponding to Figure 1, panel A2. POLK–HRP and Beta-actin blots, including molecular weight markers (Dual Color Standards; Bio-Rad, Cat. #1610374), from whole-cell lysates of mouse primary cortical neuronal cultures treated with siControl and siPOLK.
