# Supplementary figures and images for: An altered cell-specific subcellular distribution of translesion synthesis DNA polymerase kappa (POLK) in aging mouse neurons
Source: eLife. 2026 May 13;13:RP101533. doi: 10.7554/eLife.101533 (PMC13171111; doi:10.7554/eLife.101533)

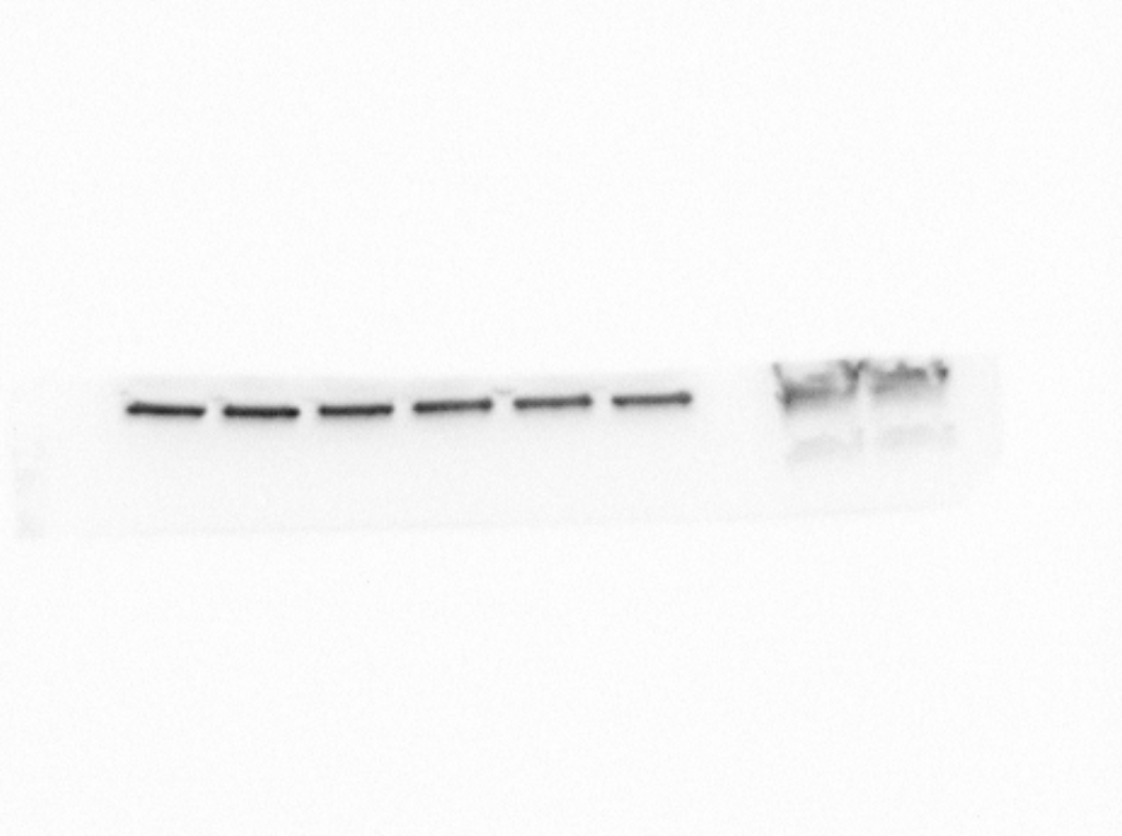

Supplement: Figure 1—source data 2. [file elife-101533-fig1-data2.zip › Figure 1_Source Data 2/Figure-1(A2)_Beta actin.tif]

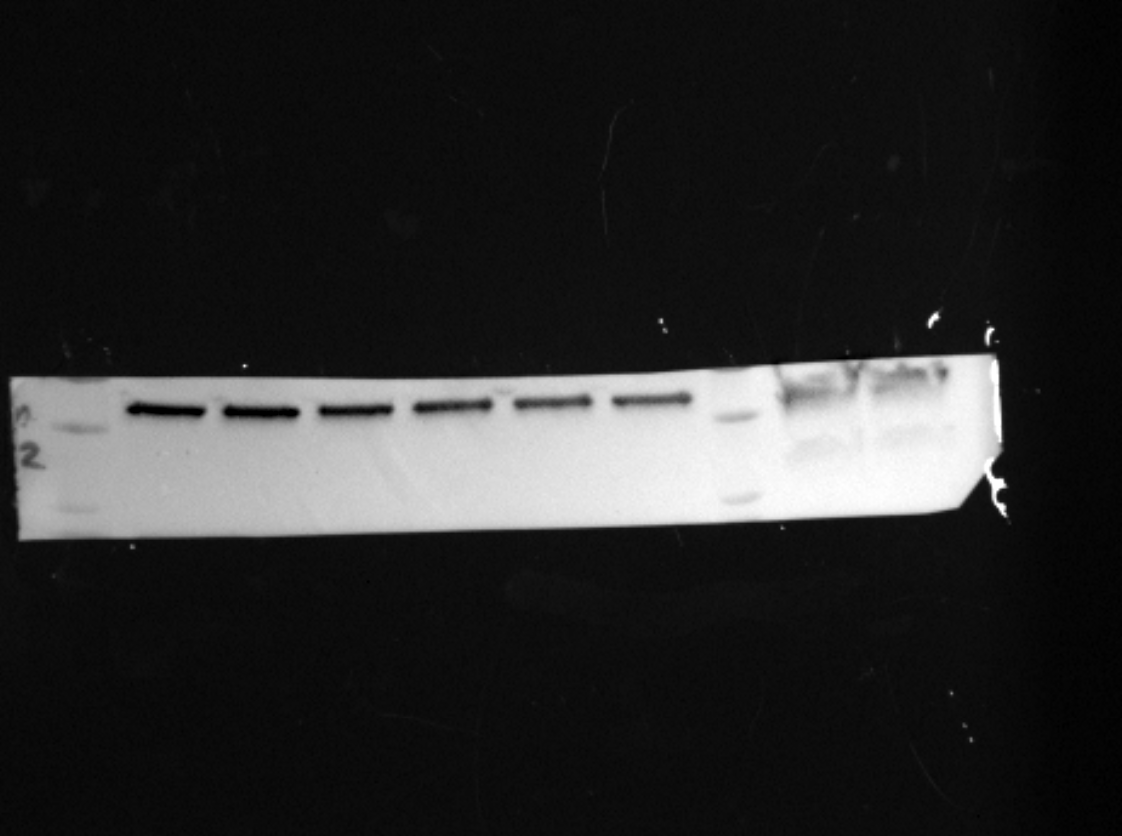

Supplement: Figure 1—source data 2. [file elife-101533-fig1-data2.zip › Figure 1_Source Data 2/Figure-1(A2)_Beta actin_Merged_with_Protein_Ladder.tif]

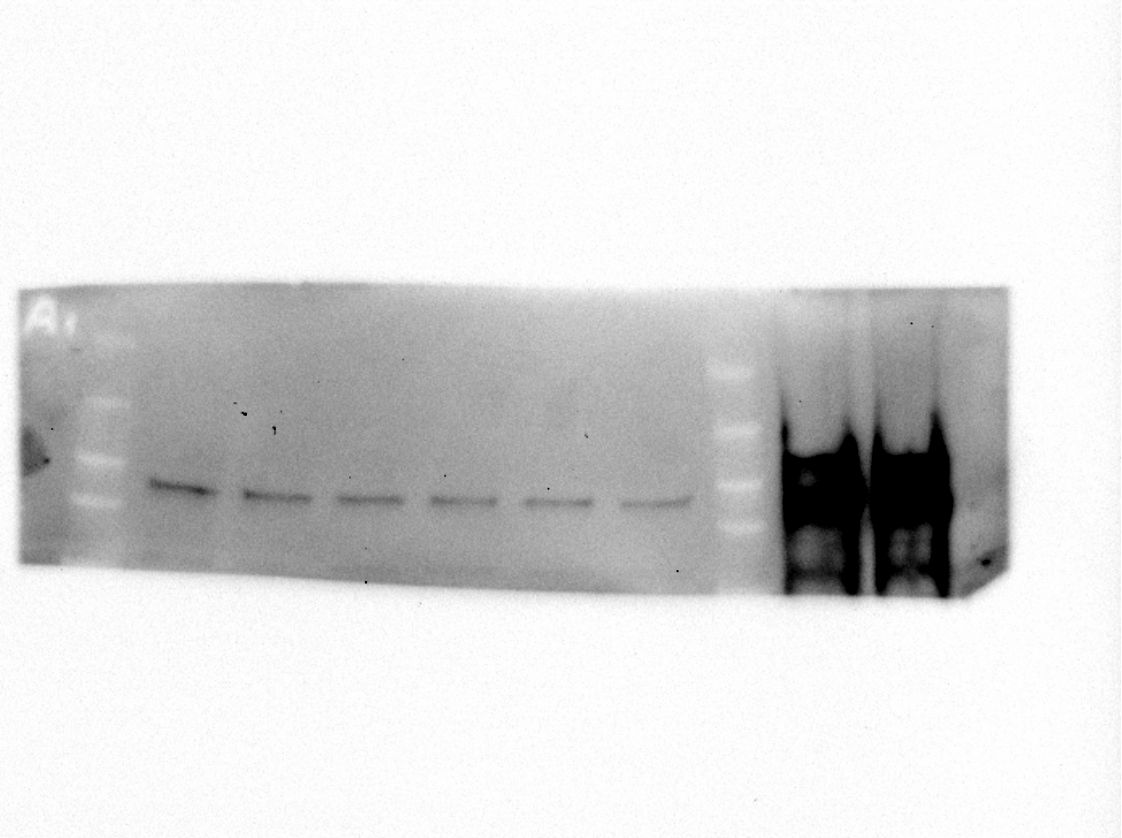

Supplement: Figure 1—source data 2. [file elife-101533-fig1-data2.zip › Figure 1_Source Data 2/Figure-1(A2)_POLK-HRP.tif]

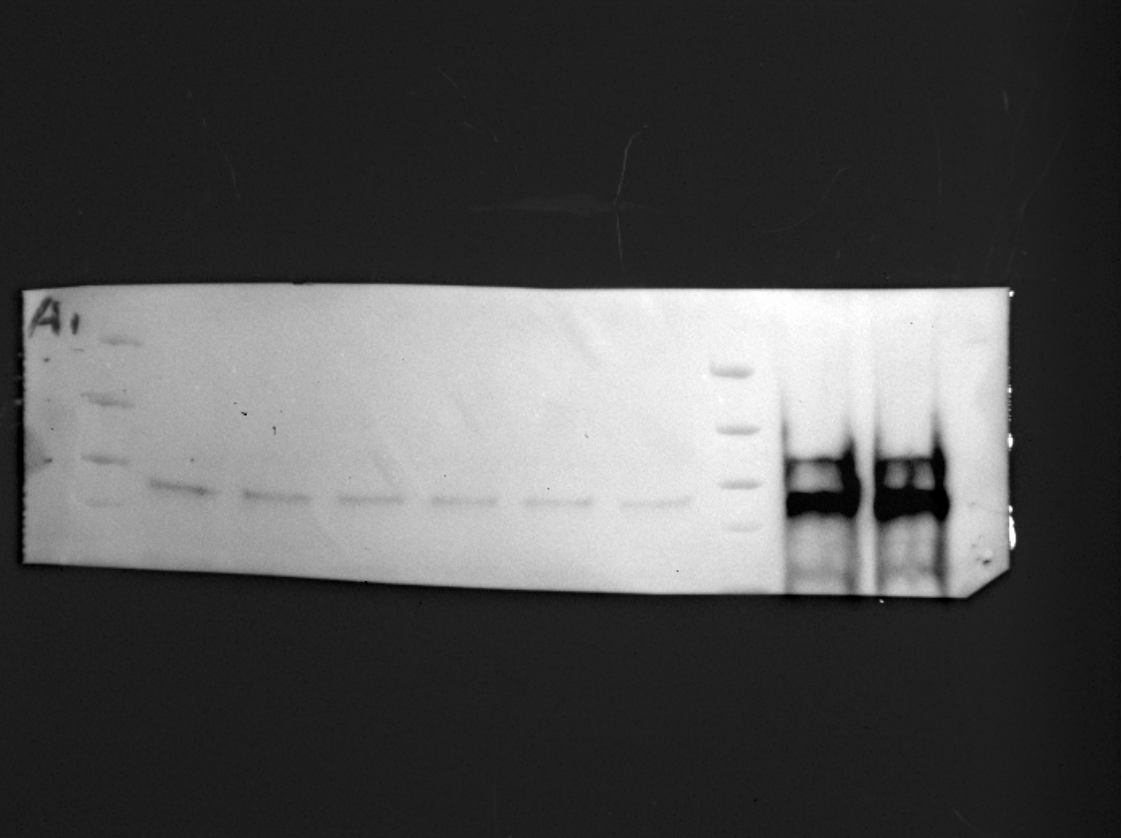

Supplement: Figure 1—source data 2. [file elife-101533-fig1-data2.zip › Figure 1_Source Data 2/Figure-1(A2)_POLK-HRP_Merged_with_Protein_Ladder.tif]

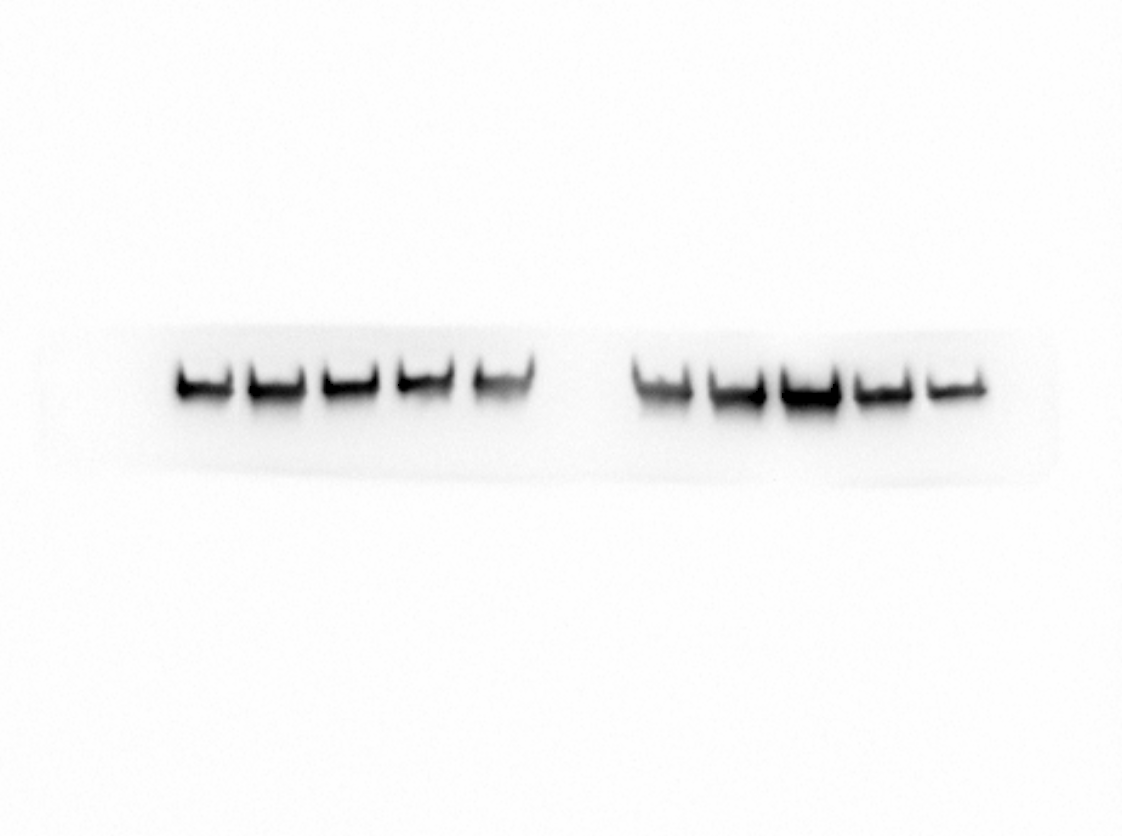

Supplement: Figure 1—figure supplement 1—source data 2. [file elife-101533-fig1-figsupp1-data2.zip › Figure 1-figure supplement 1-source data 2/Figure S1C/Figure-S1C_Beta actin.tif]

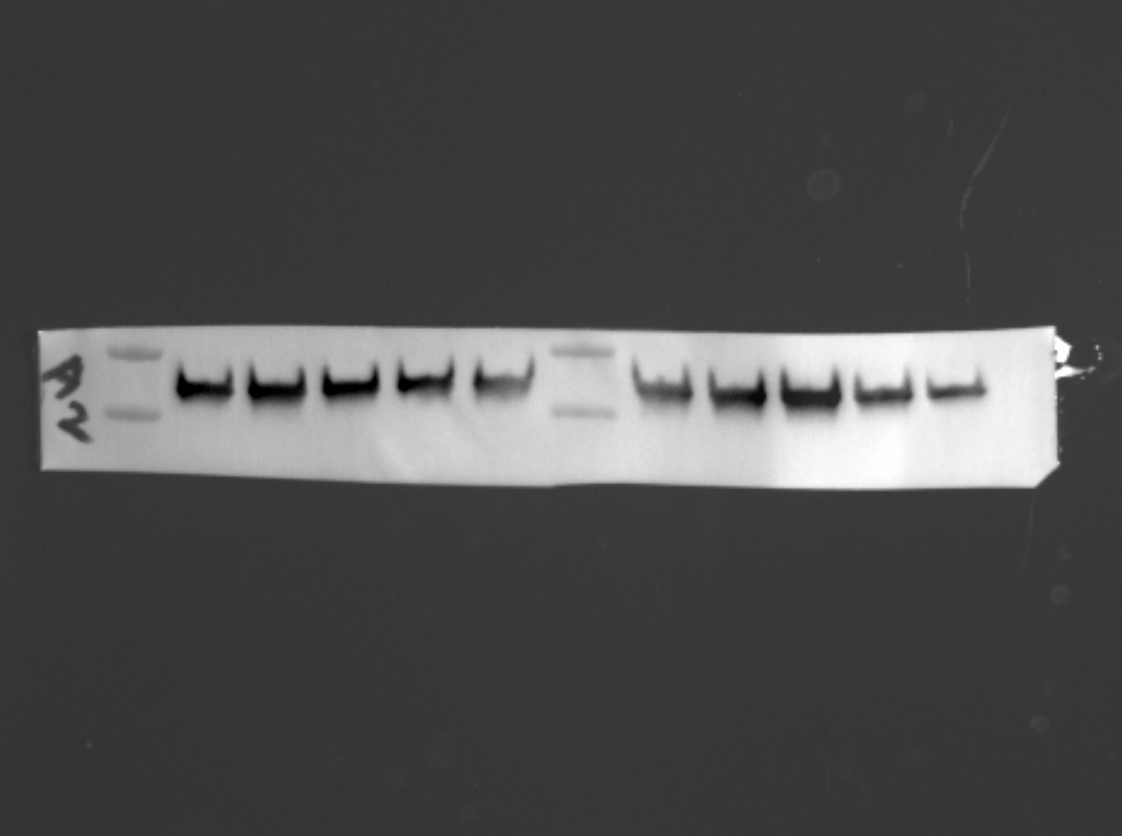

Supplement: Figure 1—figure supplement 1—source data 2. [file elife-101533-fig1-figsupp1-data2.zip › Figure 1-figure supplement 1-source data 2/Figure S1C/Figure-S1C_Beta actin_Merged_with_Protein_Ladder.tif]

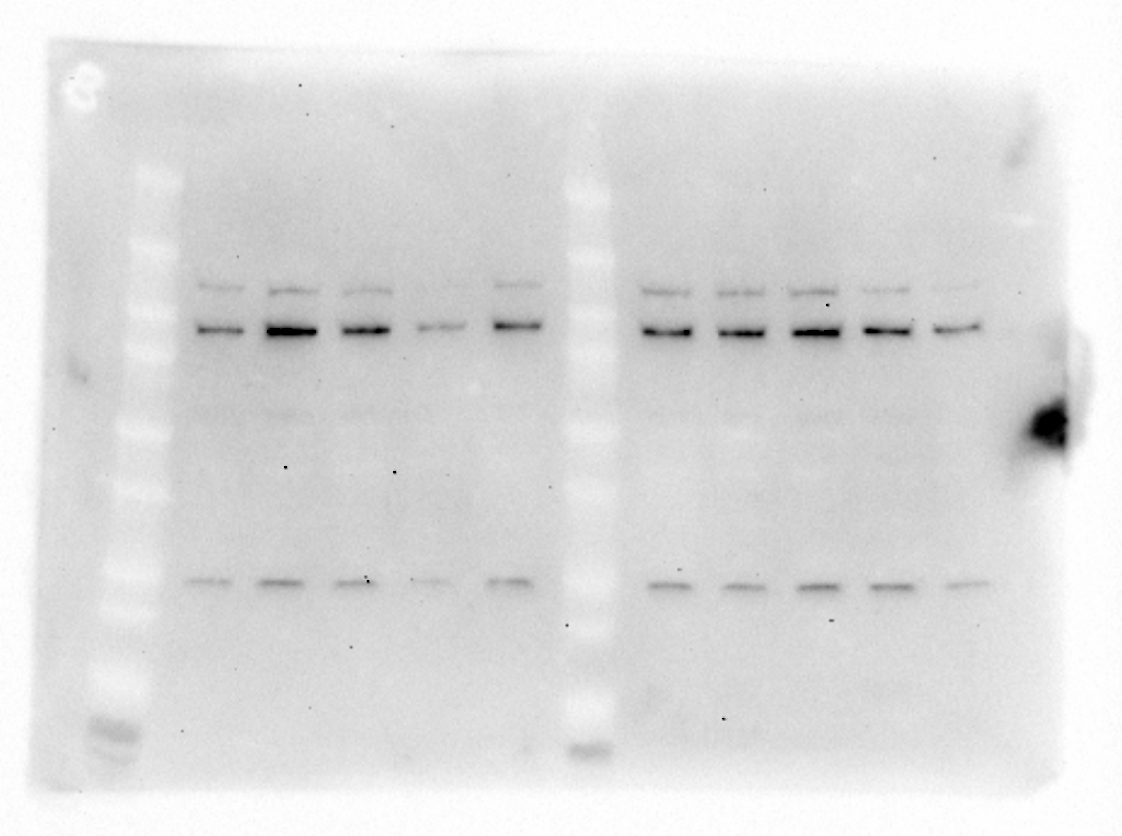

Supplement: Figure 1—figure supplement 1—source data 2. [file elife-101533-fig1-figsupp1-data2.zip › Figure 1-figure supplement 1-source data 2/Figure S1C/Figure-S1C_POLK-HRP.tif]

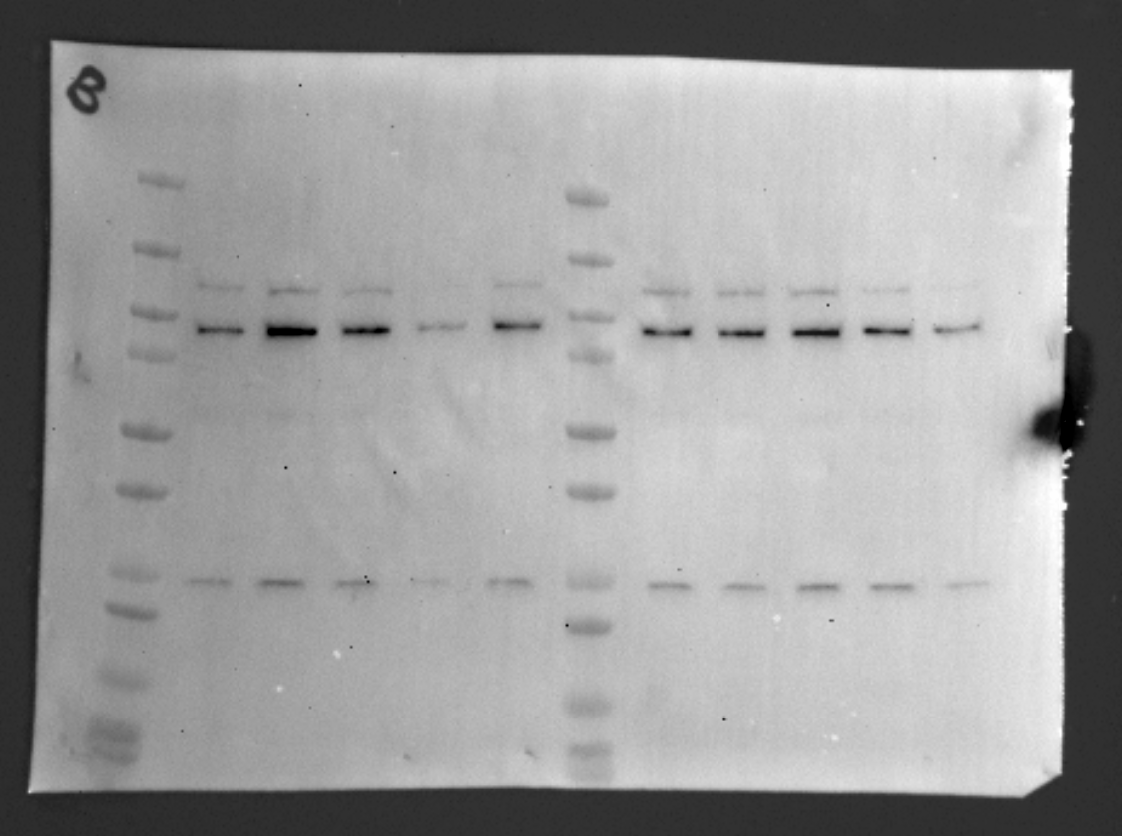

Supplement: Figure 1—figure supplement 1—source data 2. [file elife-101533-fig1-figsupp1-data2.zip › Figure 1-figure supplement 1-source data 2/Figure S1C/Figure-S1C_POLK-HRP_Merged_with_Protein_Ladder.tif]

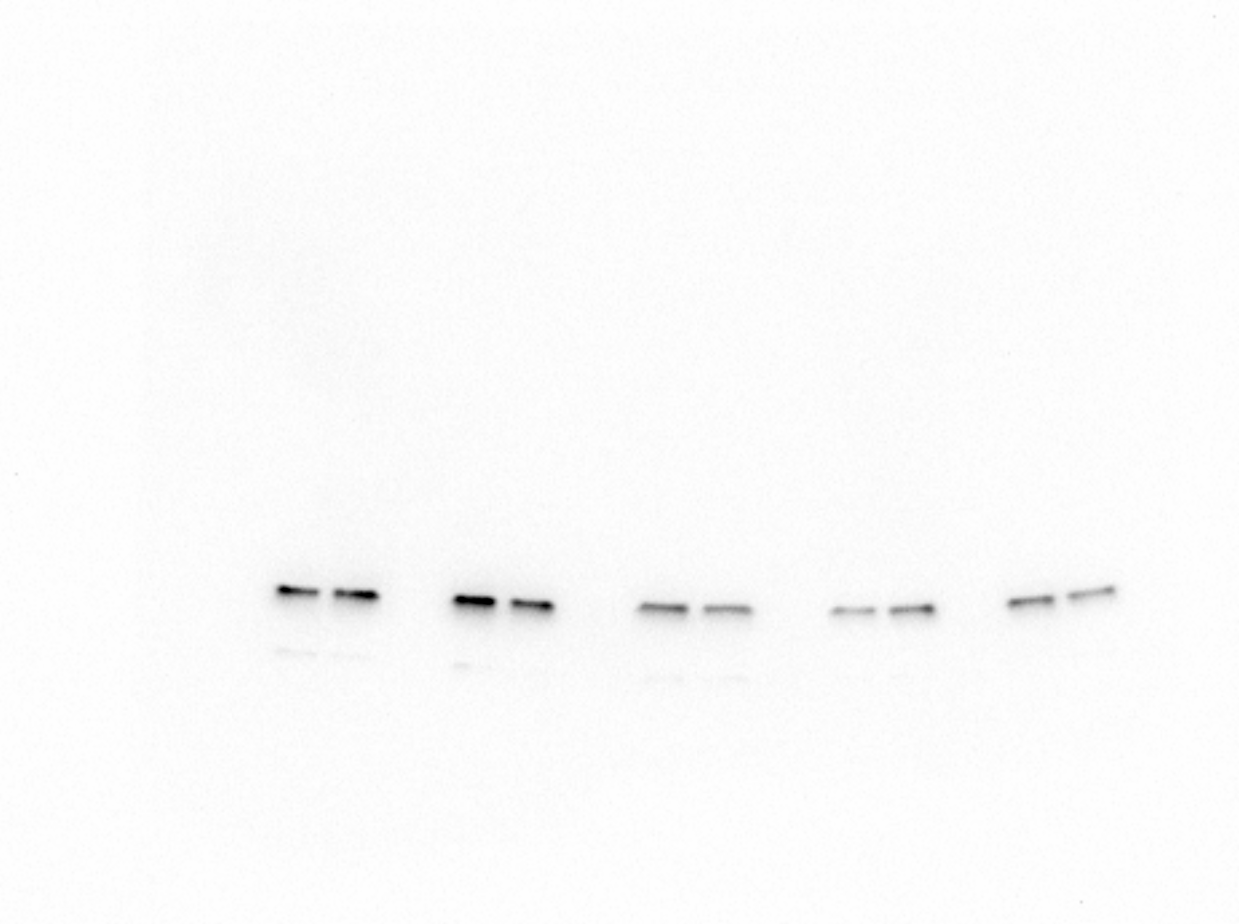

Supplement: Figure 1—figure supplement 1—source data 2. [file elife-101533-fig1-figsupp1-data2.zip › Figure 1-figure supplement 1-source data 2/Figure-S1D/Figure-S1D_GAPDH.tif]

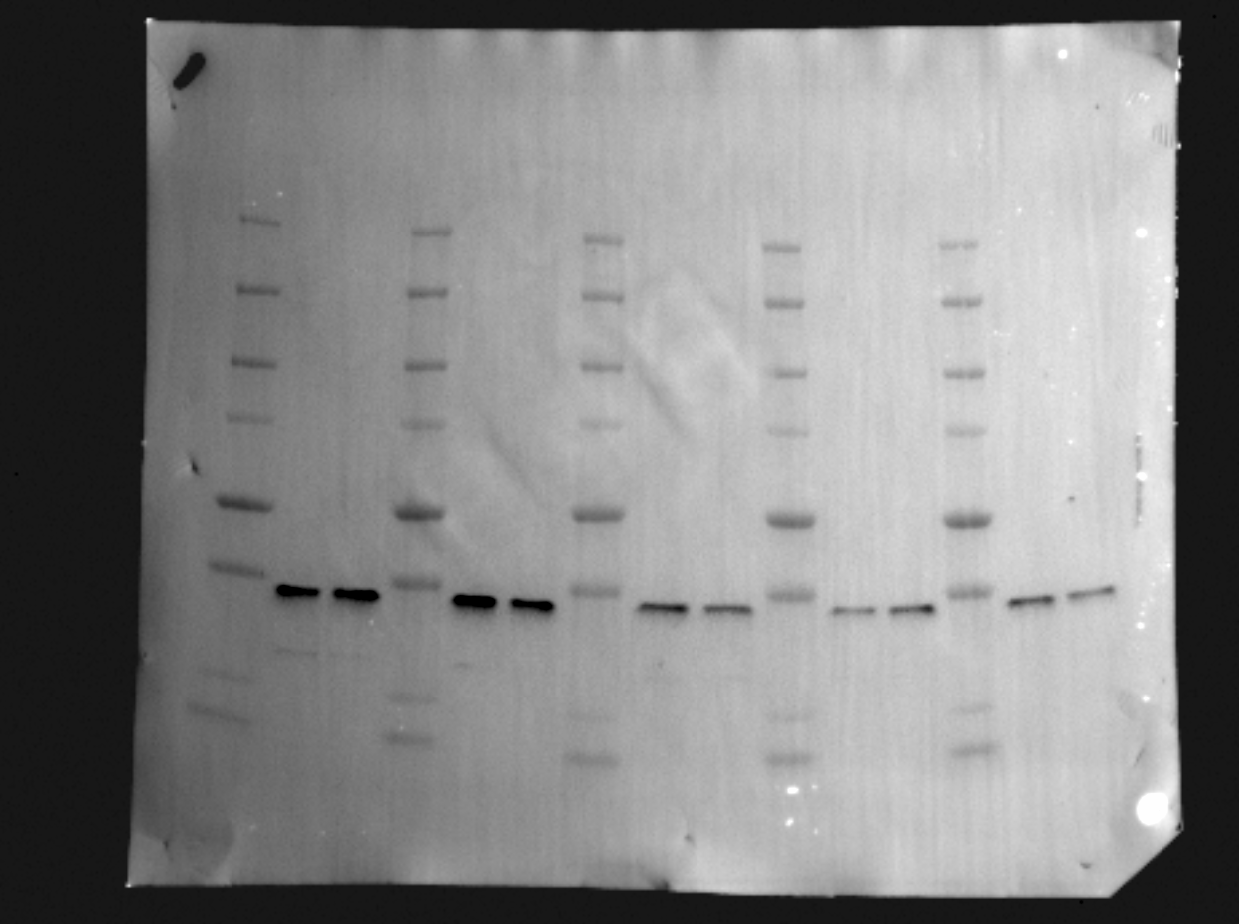

Supplement: Figure 1—figure supplement 1—source data 2. [file elife-101533-fig1-figsupp1-data2.zip › Figure 1-figure supplement 1-source data 2/Figure-S1D/Figure-S1D_GAPDH_Merged_with_Protein_Ladder.tif]

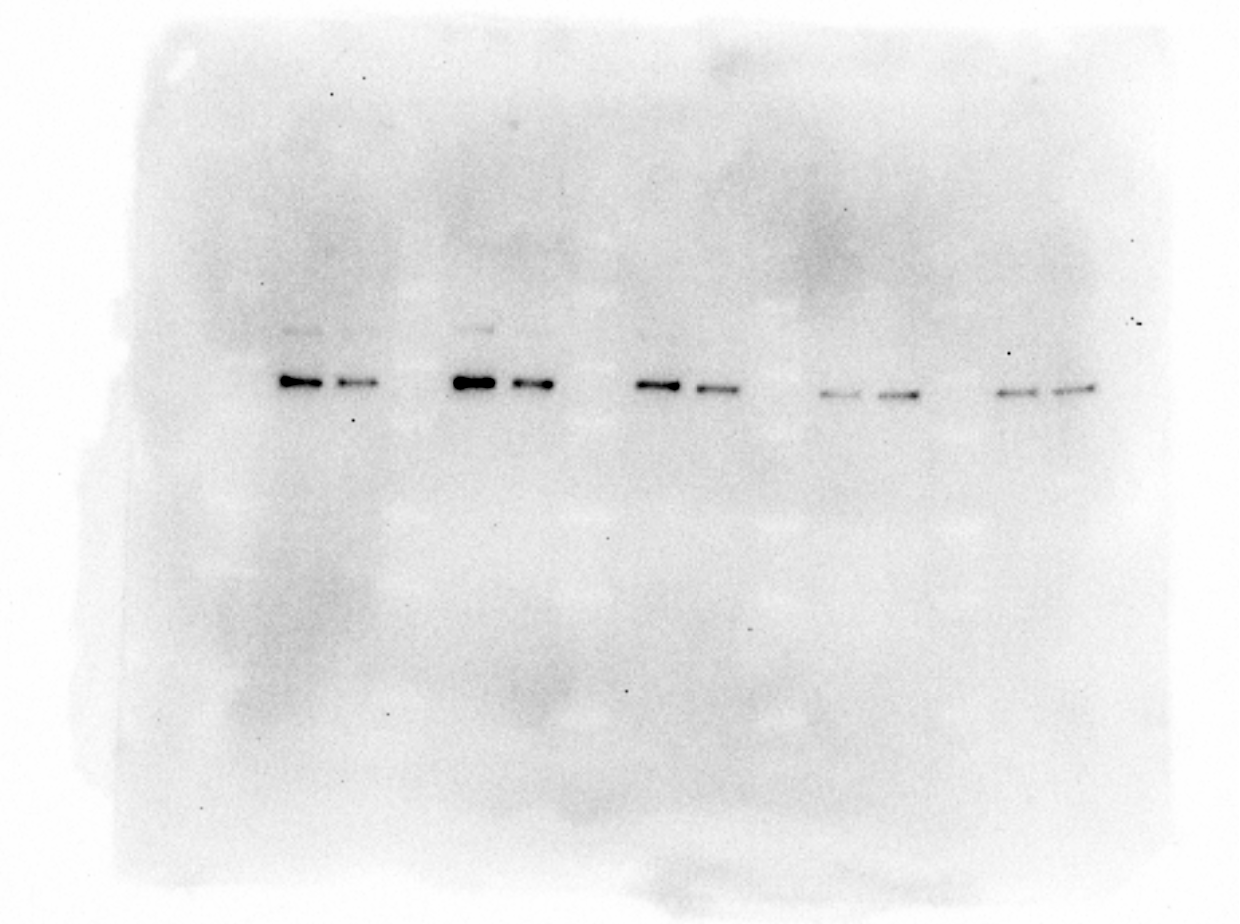

Supplement: Figure 1—figure supplement 1—source data 2. [file elife-101533-fig1-figsupp1-data2.zip › Figure 1-figure supplement 1-source data 2/Figure-S1D/Figure-S1D_POLK-HRP.tif]

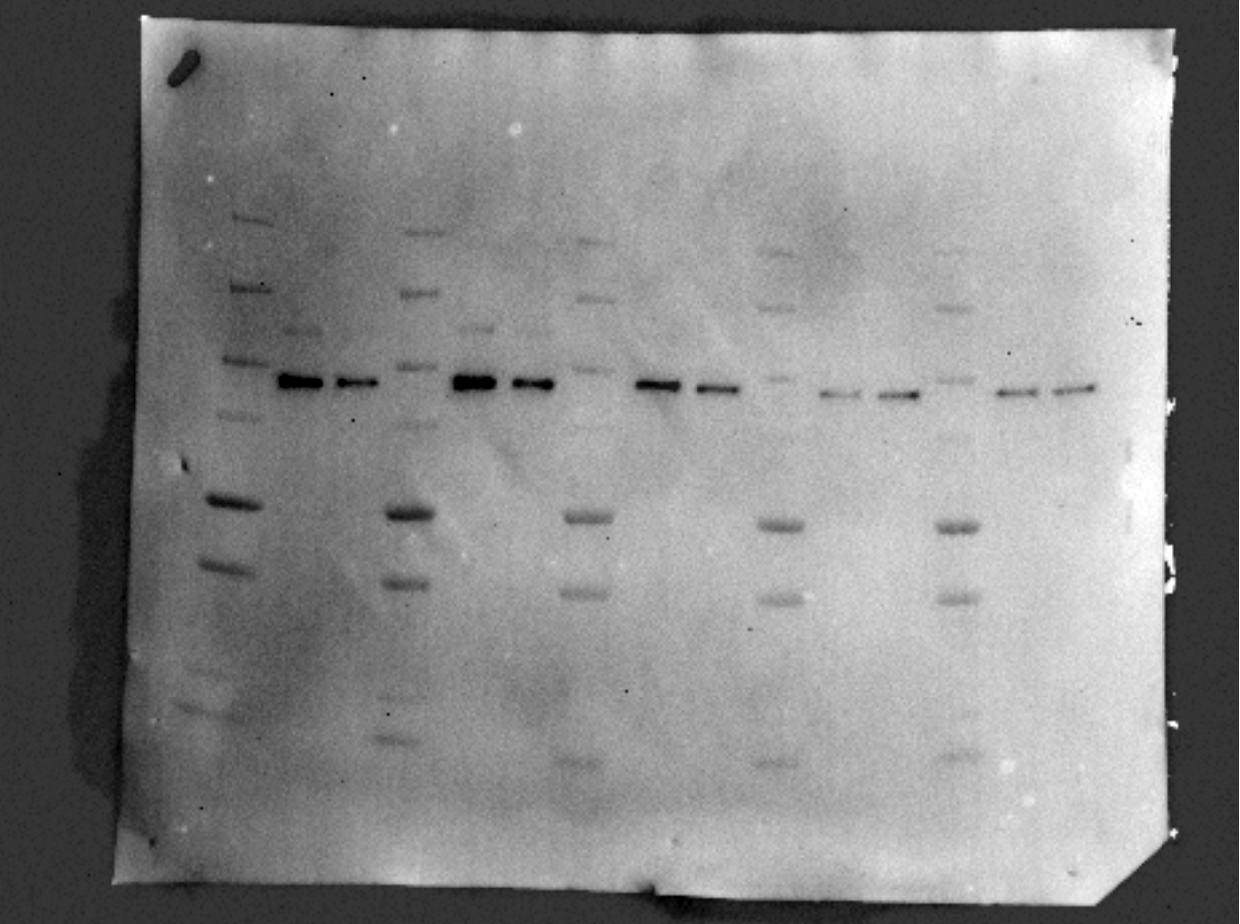

Supplement: Figure 1—figure supplement 1—source data 2. [file elife-101533-fig1-figsupp1-data2.zip › Figure 1-figure supplement 1-source data 2/Figure-S1D/Figure-S1D_POLK-HRP_Merged_with_Protein_Ladder.tif]

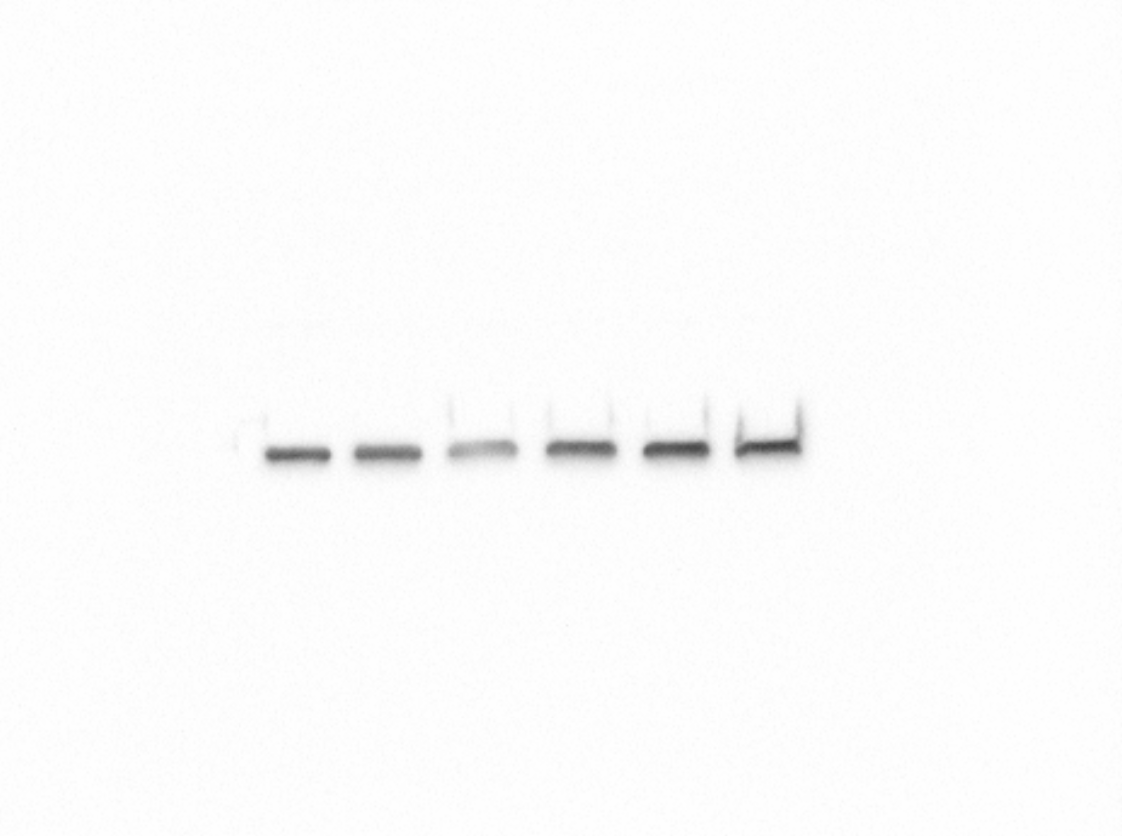

Supplement: Figure 1—figure supplement 1—source data 2. [file elife-101533-fig1-figsupp1-data2.zip › Figure 1-figure supplement 1-source data 2/Figure-S1E/Figure-S1E_Beta actin.tif]

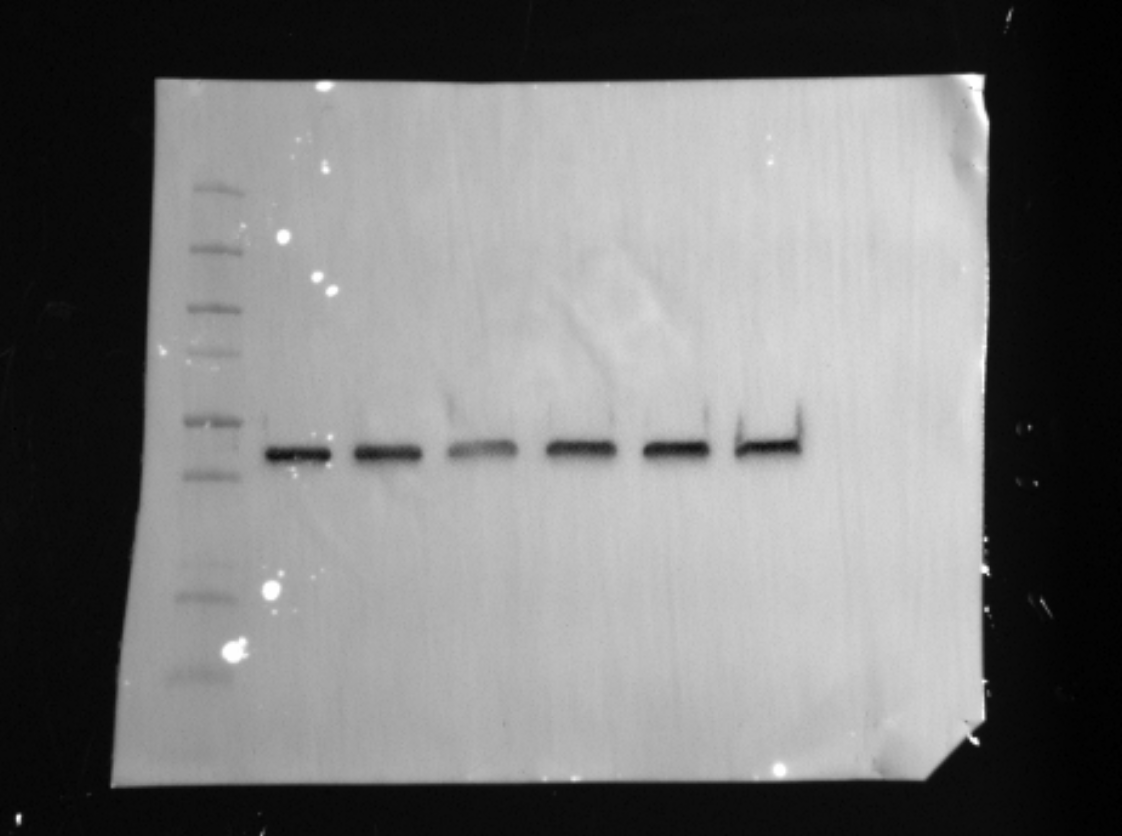

Supplement: Figure 1—figure supplement 1—source data 2. [file elife-101533-fig1-figsupp1-data2.zip › Figure 1-figure supplement 1-source data 2/Figure-S1E/Figure-S1E_Beta actin_Merged_with_Protein_Ladder.tif]

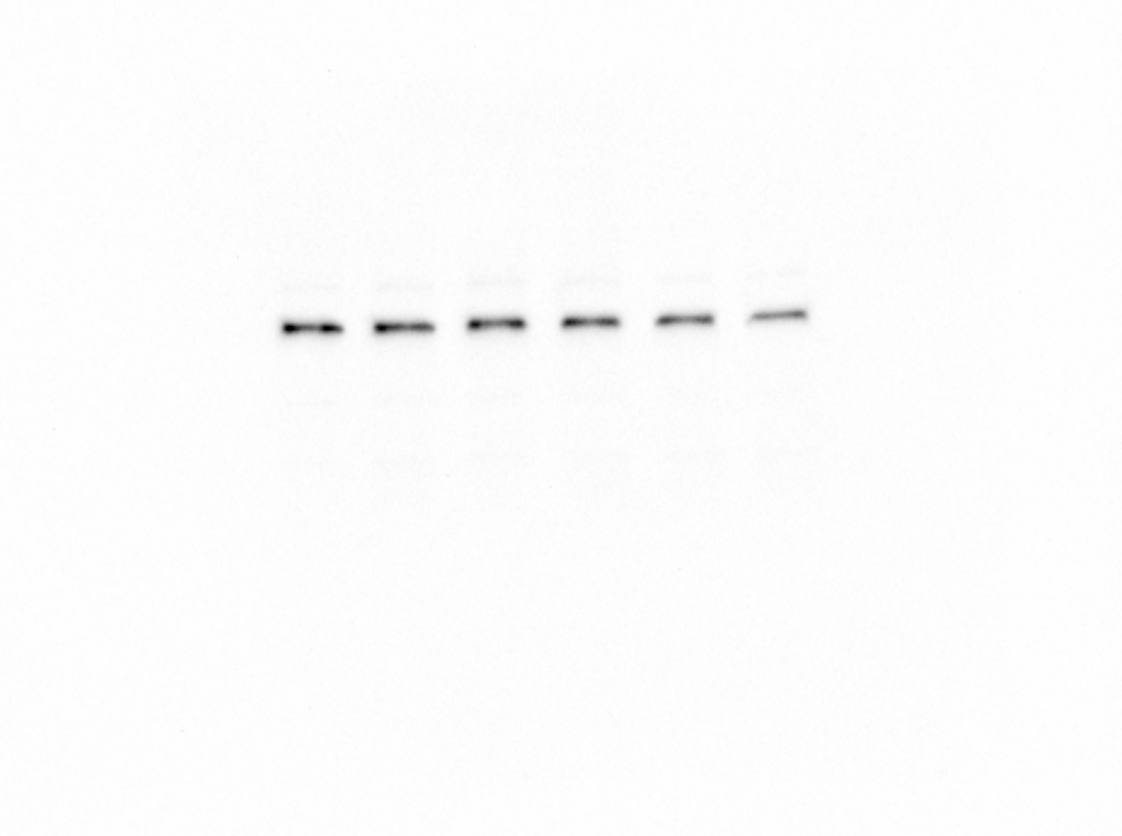

Supplement: Figure 1—figure supplement 1—source data 2. [file elife-101533-fig1-figsupp1-data2.zip › Figure 1-figure supplement 1-source data 2/Figure-S1E/Figure-S1E_POLK-HRP.tif]

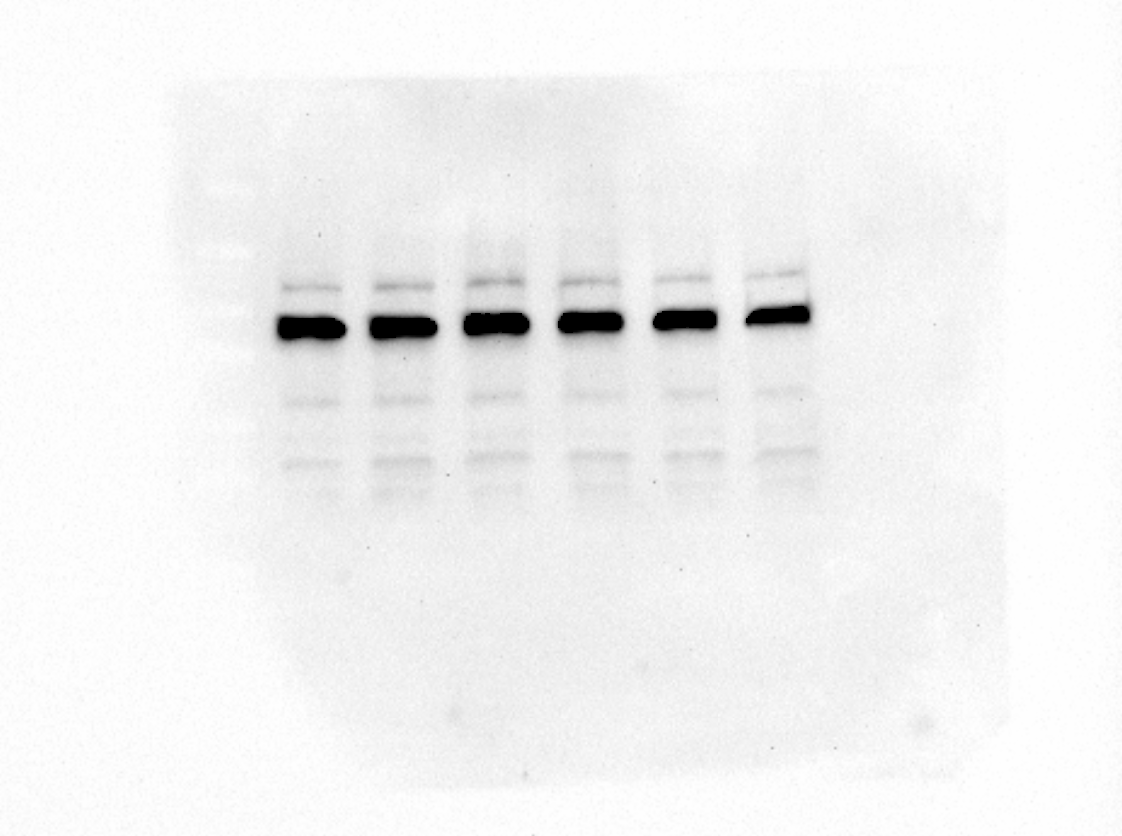

Supplement: Figure 1—figure supplement 1—source data 2. [file elife-101533-fig1-figsupp1-data2.zip › Figure 1-figure supplement 1-source data 2/Figure-S1E/Figure-S1E_POLK-HRP_High-Exposure.tif]

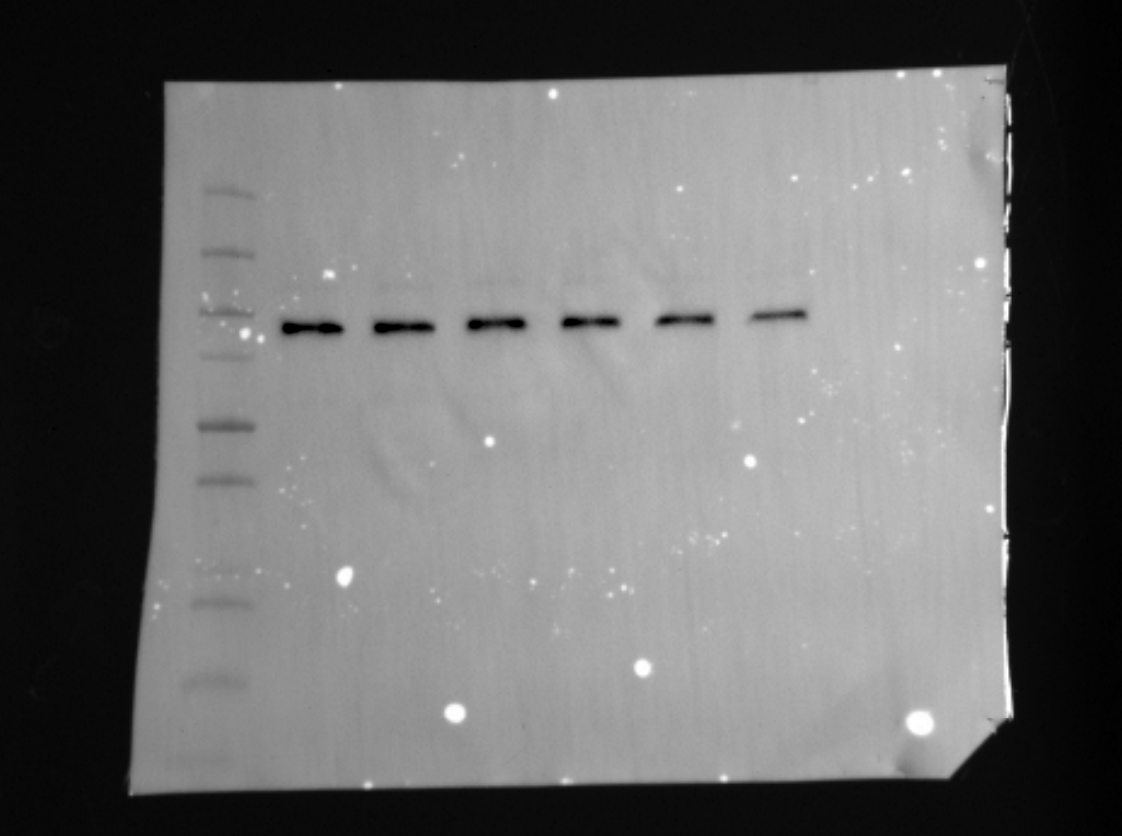

Supplement: Figure 1—figure supplement 1—source data 2. [file elife-101533-fig1-figsupp1-data2.zip › Figure 1-figure supplement 1-source data 2/Figure-S1E/Figure-S1E_POLK-HRP_Merged_with_Protein_Ladder.tif]

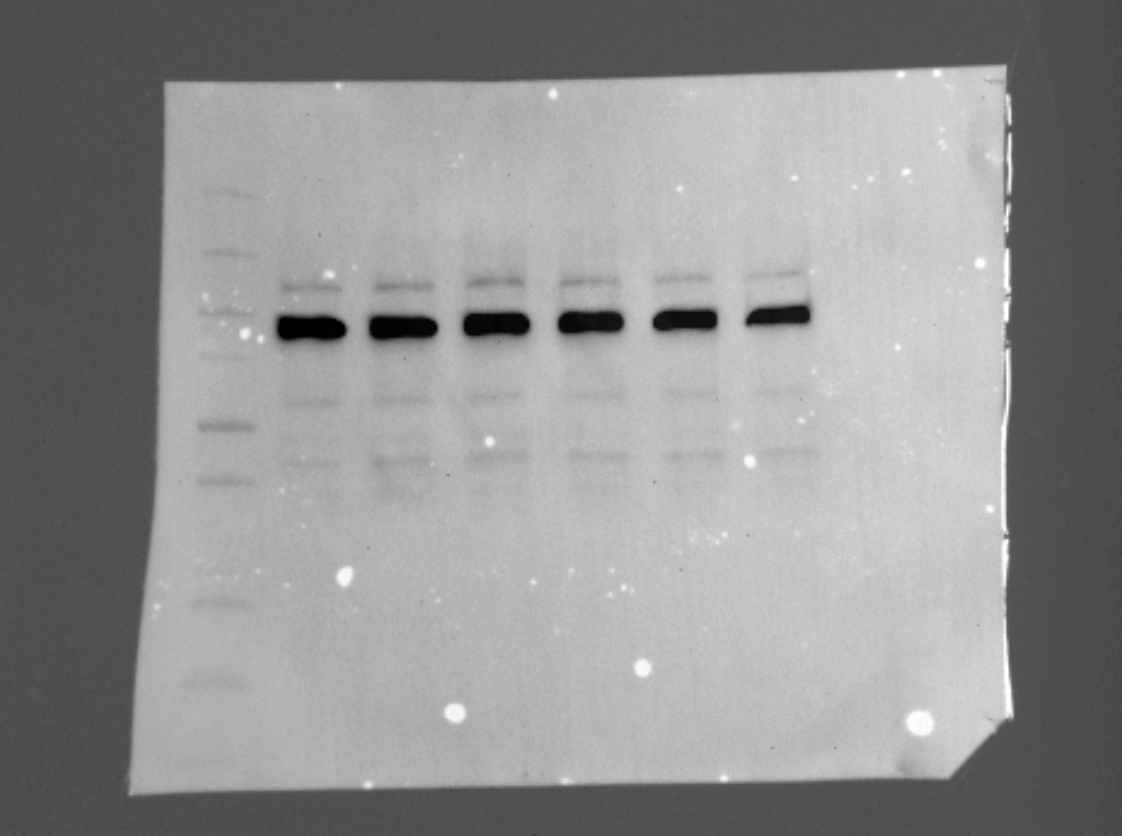

Supplement: Figure 1—figure supplement 1—source data 2. [file elife-101533-fig1-figsupp1-data2.zip › Figure 1-figure supplement 1-source data 2/Figure-S1E/Figure-S1E_POLK-HRP_Merged_with_Protein_Ladder_High-Exposure.tif]

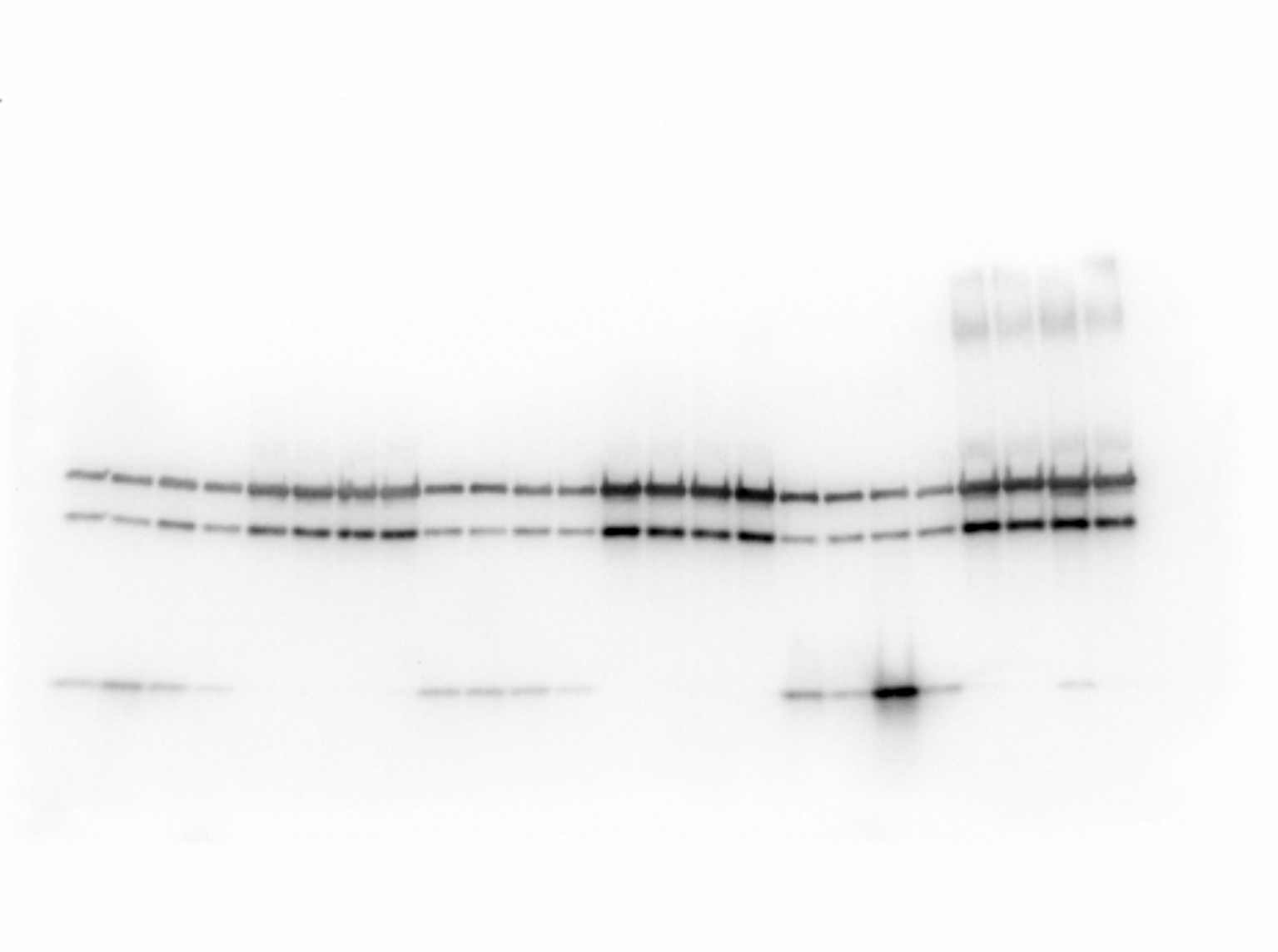

Supplement: Figure 1—figure supplement 1—source data 2. [file elife-101533-fig1-figsupp1-data2.zip › Figure 1-figure supplement 1-source data 2/Figure-S1G/Figure-S1G_GAPDH.tif]

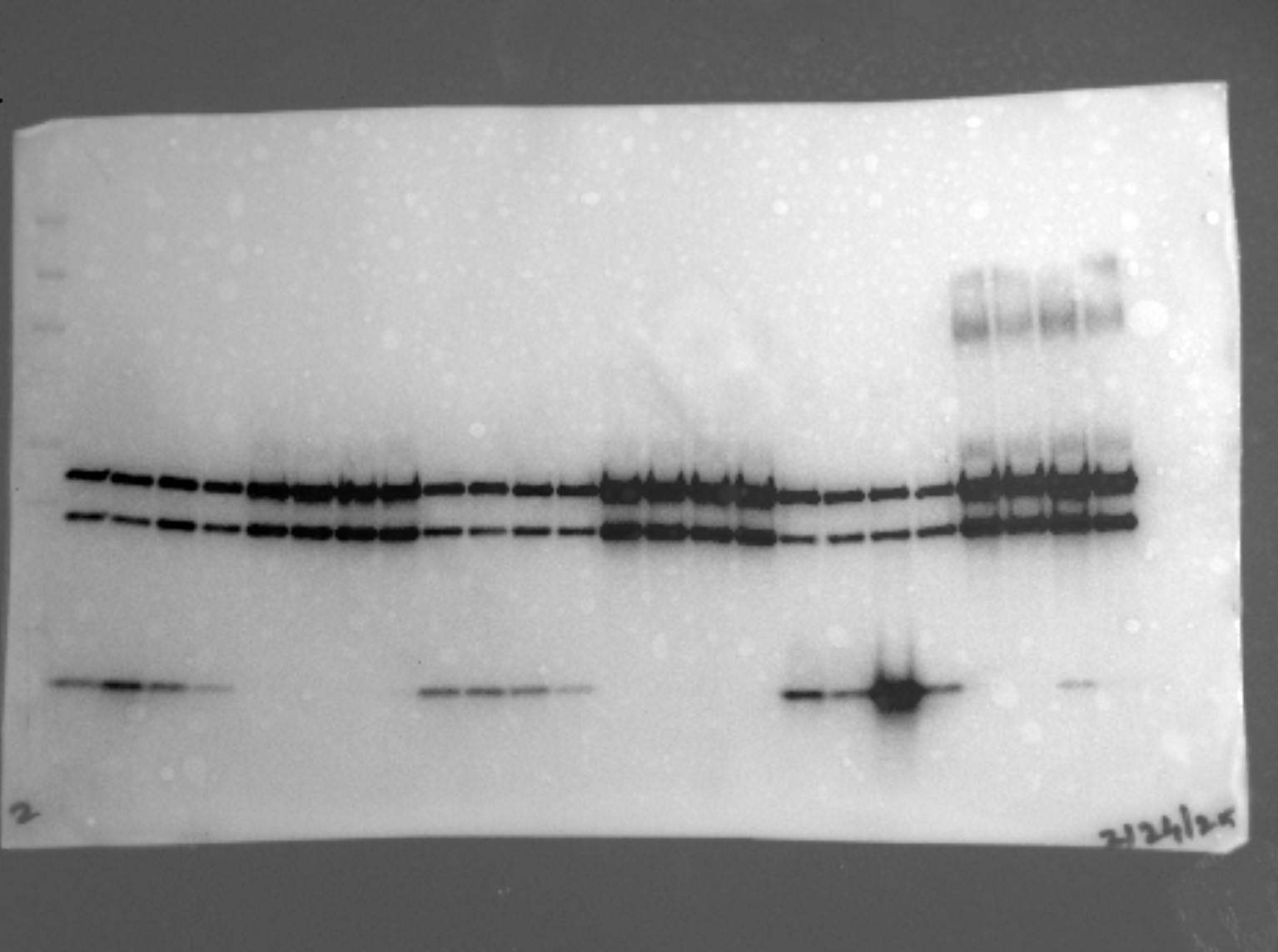

Supplement: Figure 1—figure supplement 1—source data 2. [file elife-101533-fig1-figsupp1-data2.zip › Figure 1-figure supplement 1-source data 2/Figure-S1G/Figure-S1G_GAPDH_&_Histone H3_Merged_with_Protein_Ladder.tif]

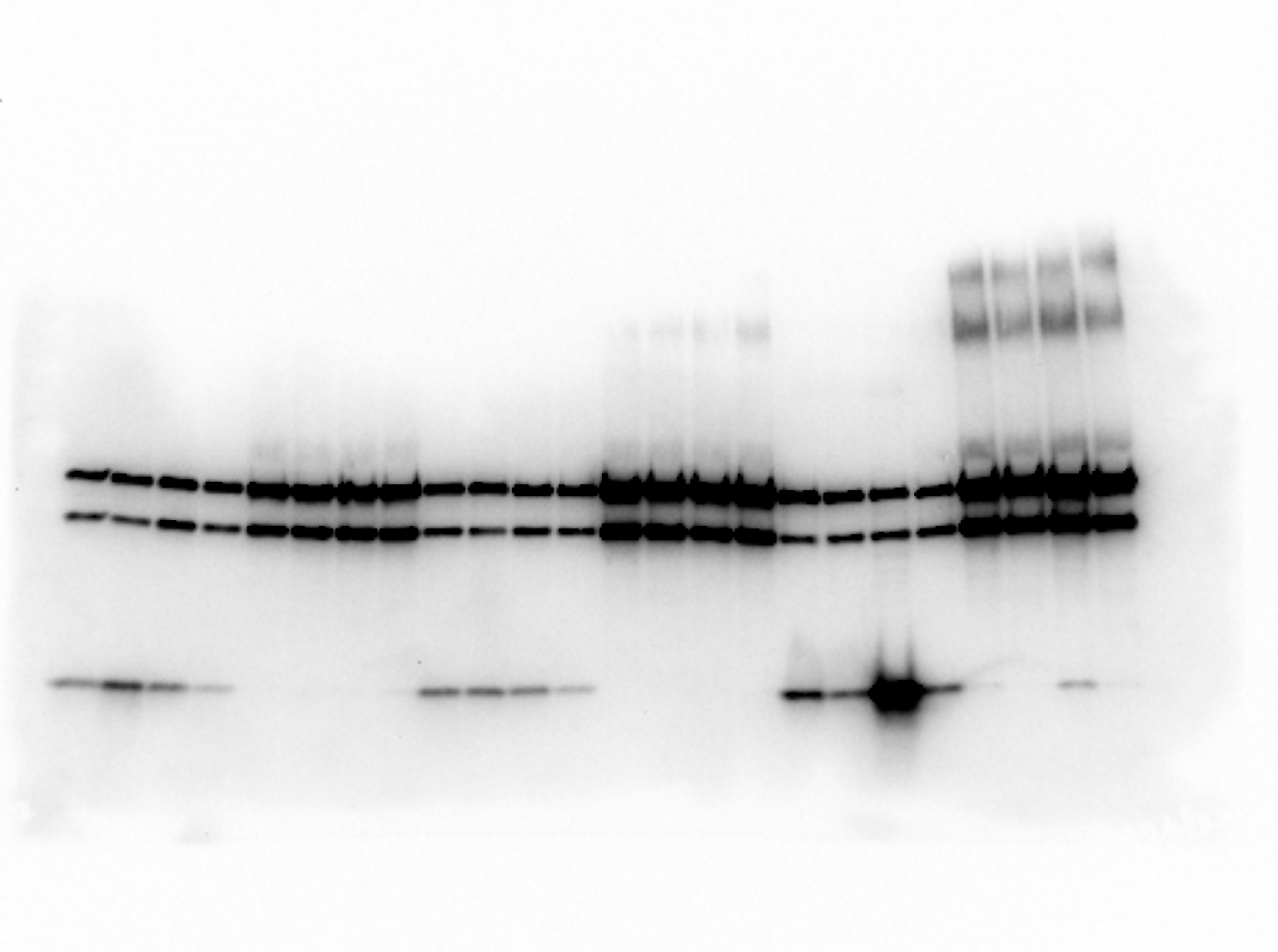

Supplement: Figure 1—figure supplement 1—source data 2. [file elife-101533-fig1-figsupp1-data2.zip › Figure 1-figure supplement 1-source data 2/Figure-S1G/Figure-S1G_Histone H3.tif]

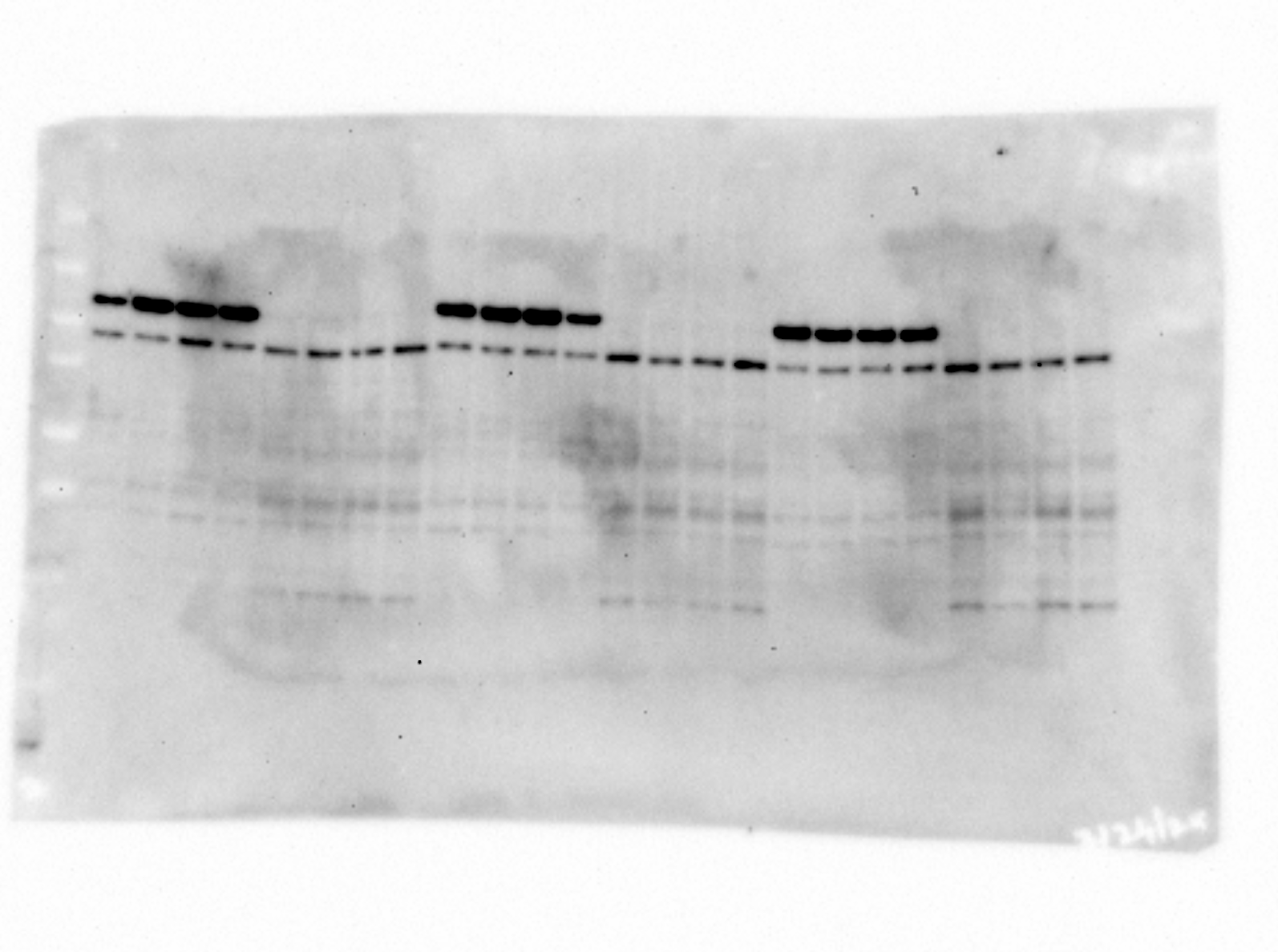

Supplement: Figure 1—figure supplement 1—source data 2. [file elife-101533-fig1-figsupp1-data2.zip › Figure 1-figure supplement 1-source data 2/Figure-S1G/Figure-S1G_POLK-HRP.tif]

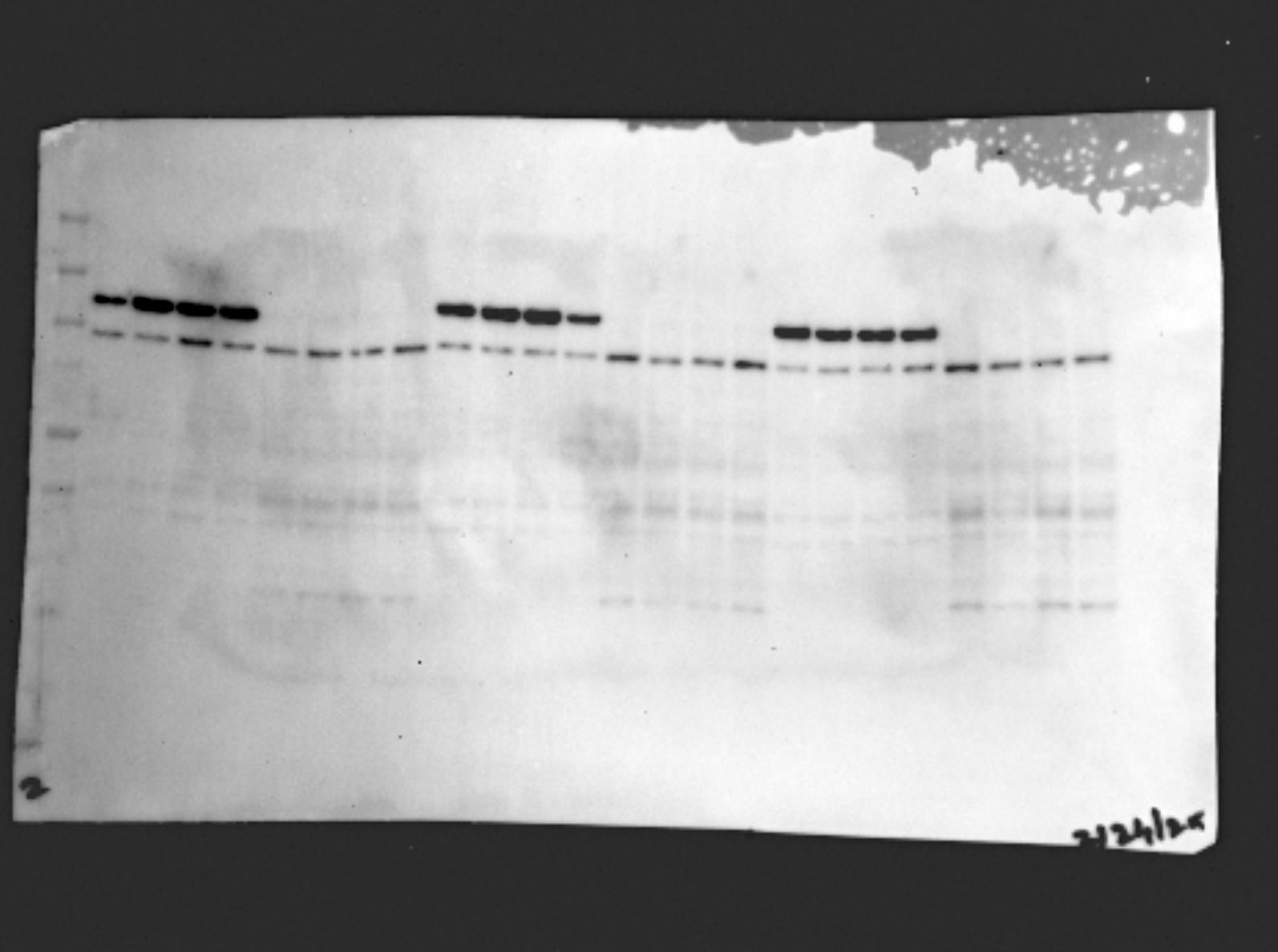

Supplement: Figure 1—figure supplement 1—source data 2. [file elife-101533-fig1-figsupp1-data2.zip › Figure 1-figure supplement 1-source data 2/Figure-S1G/Figure-S1G_POLK-HRP_Merged_with_Protein_Ladder.tif]
