# Supplementary material for: An altered cell-specific subcellular distribution of translesion synthesis DNA polymerase kappa (POLK) in aging mouse neurons
Source: eLife. 2026 May 13;13:RP101533. doi: 10.7554/eLife.101533 (PMC13171111; doi:10.7554/eLife.101533)
Supplement: Figure 1—figure supplement 1—source data 1. [file elife-101533-fig1-figsupp1-data1.zip › Figure 1-figure supplement 1-source data 1.pdf]

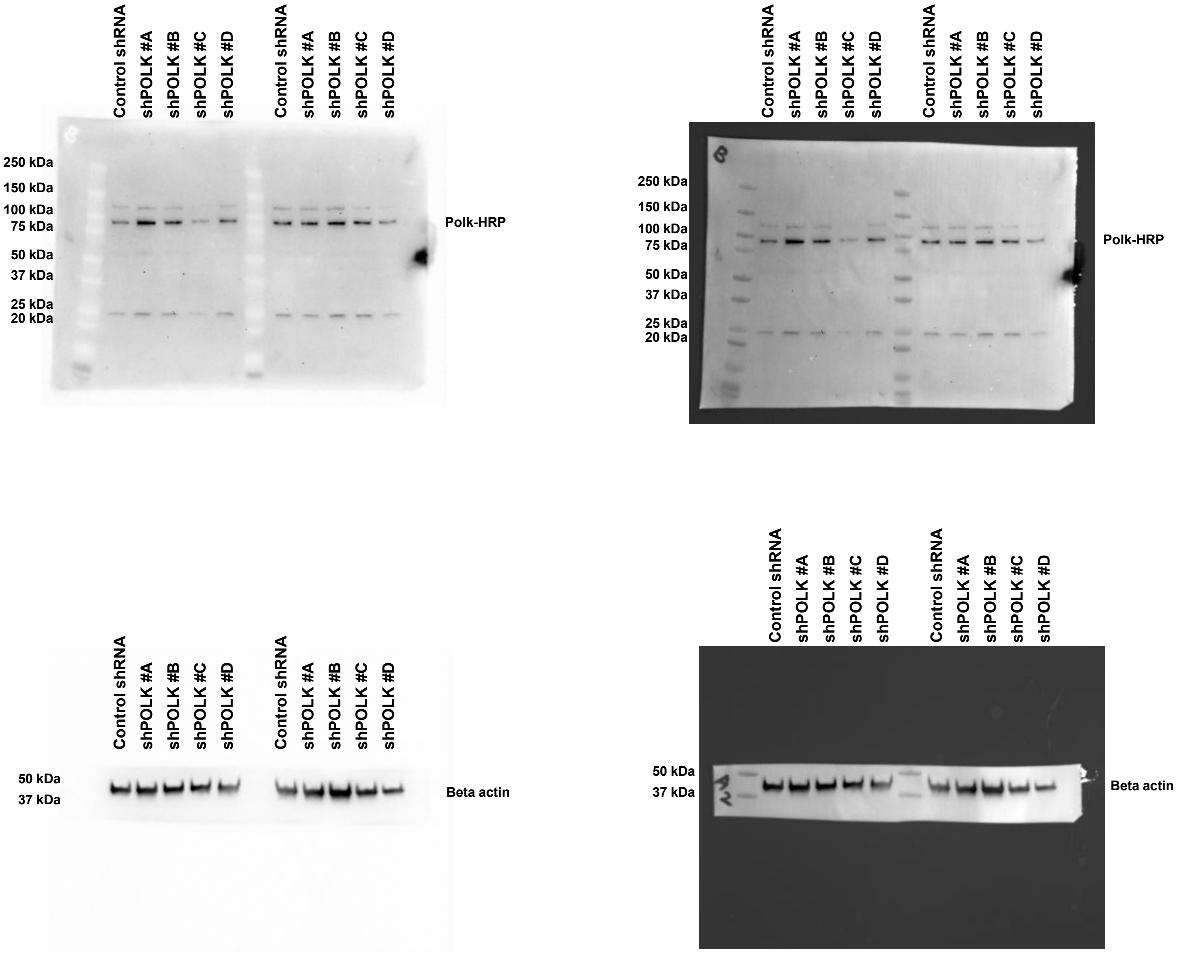

Figure 1-figure supplement 1- source data 1. Original blots corresponding to Figure 1-figure supplement 1, panel C. POLK–HRP and Beta actin blots, including molecular weight markers (Dual Color Standards; Bio-Rad, Cat. #1610374), from whole-cell lysates of mouse primary cortical neurons treated with four individual shPolk lentiviruses.

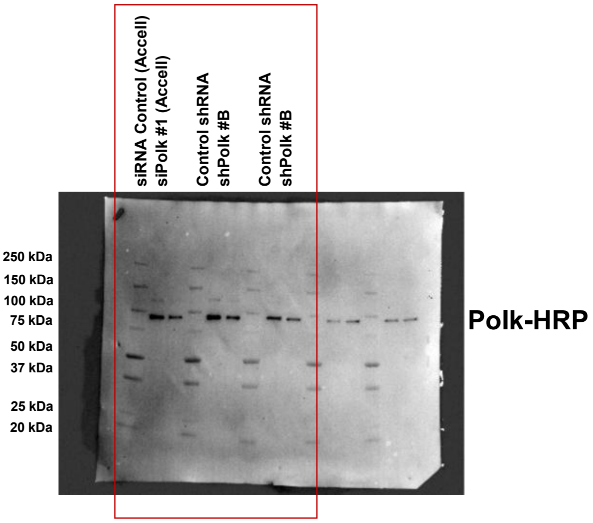

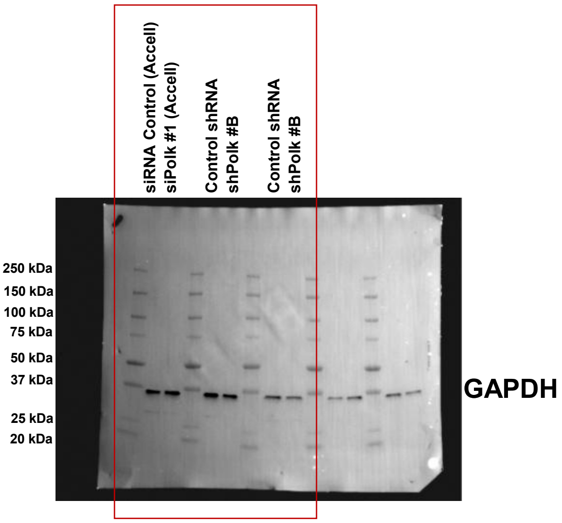

Figure 1-figure supplement 1- source data 1. Original blots corresponding to Figure 1-figure supplement 1, panel D. POLK–HRP and GAPDH blots, including molecular weight markers (Dual Color Standards; Bio-Rad, Cat. #1610374), from whole-cell lysates of mouse Neuro-2A cells treated with either siPolk or shPolk lentivirus.

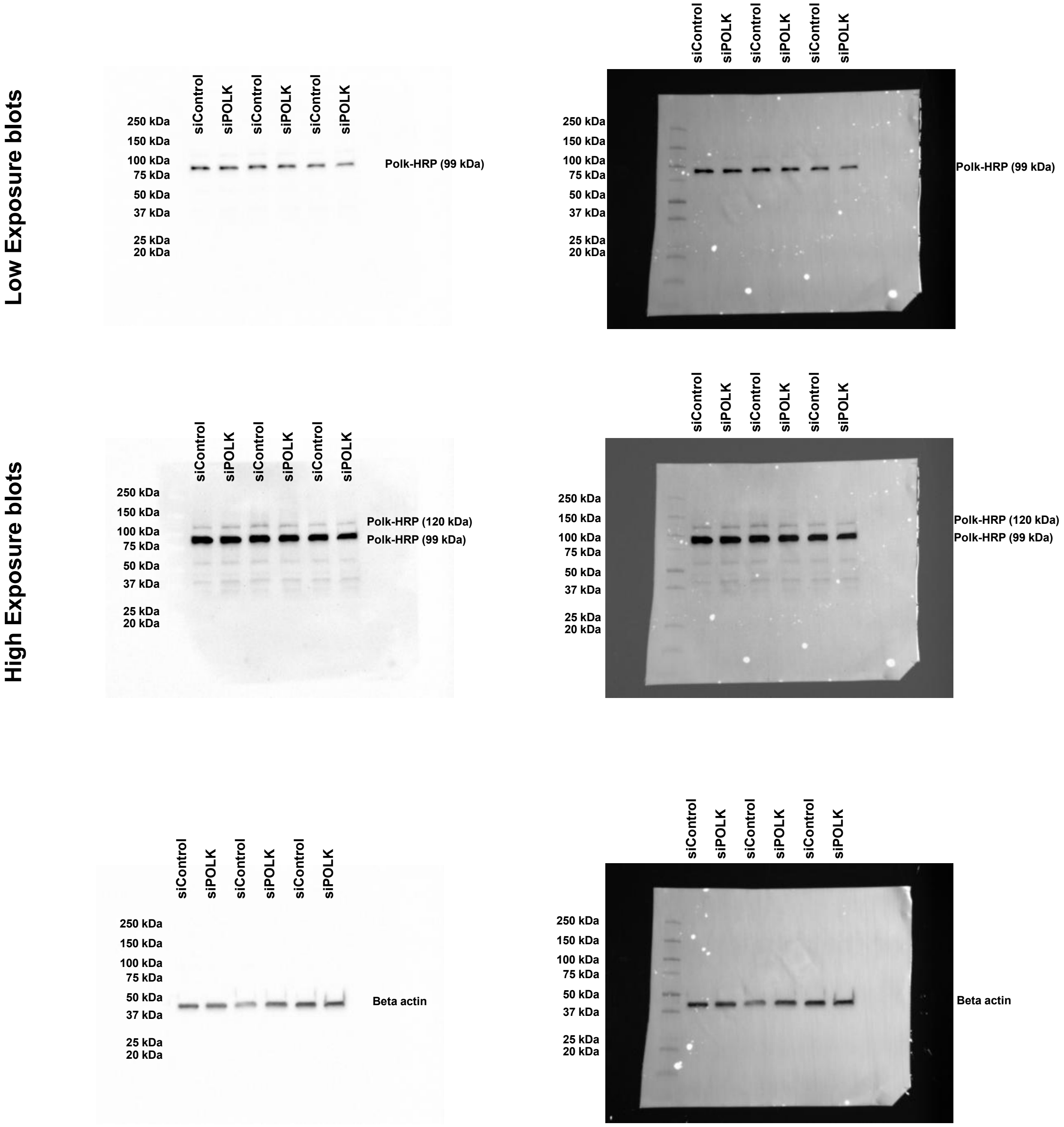

Figure 1-figure supplement 1- source data 1. Original blots corresponding to Figure 1-figure supplement 1, panel E. POLK–HRP (high- and low-exposure blots) and β-actin blots, including molecular weight markers (Dual Color Standards; Bio-Rad, Cat. #1610374), from whole-cell lysates of 4T1 cells treated with siControl and siPolk.

Mouse whole brain without cerebellum samples

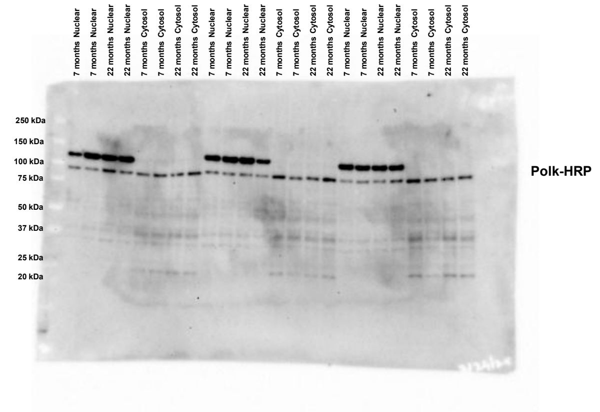

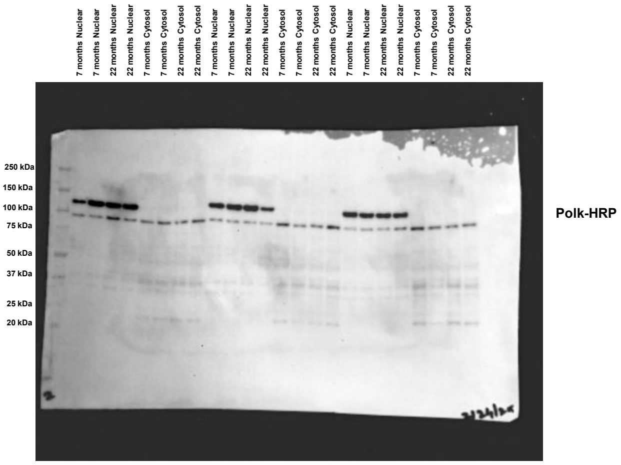

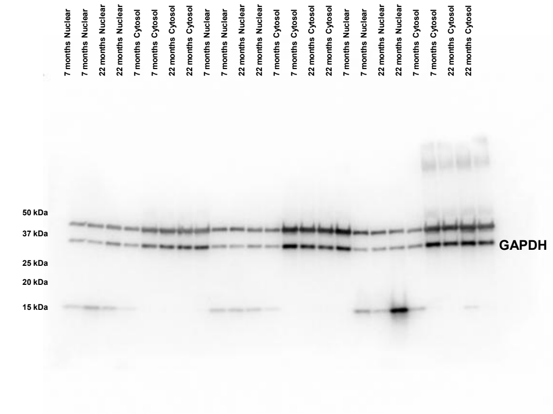

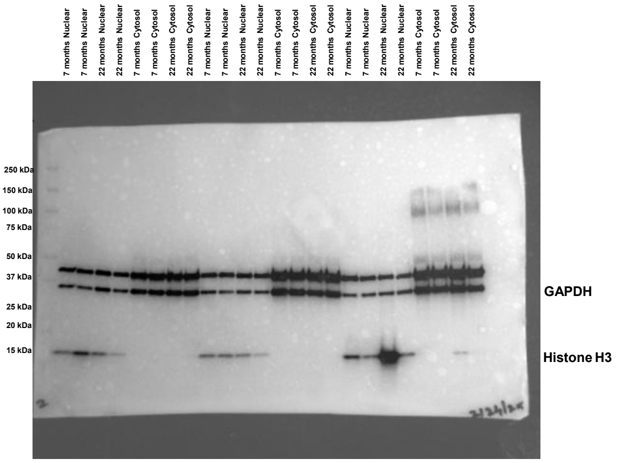

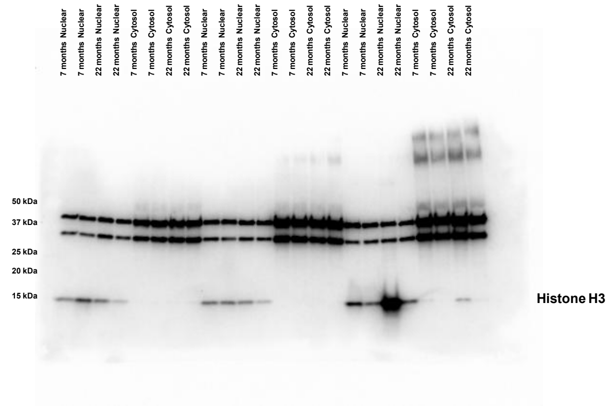

Figure 1-figure supplement 1- source data 1. Original blots corresponding to Figure 1-figure supplement 1, panel G. POLK–HRP, GAPDH, and histone H3 blots, including molecular weight markers (Dual Color Standards; Bio-Rad, Cat. #1610374), from nuclear and cytosolic fractions of whole mouse brain samples (cerebellum excluded).
